# Does Ad Libitum Feeding during the Peri-Partum Improve the Sow Feed Intake and Performances?

**DOI:** 10.3390/ani9121078

**Published:** 2019-12-03

**Authors:** Laura Martí, María Ángeles Latorre, Javier Álvarez-Rodríguez

**Affiliations:** 1 Departamento de Ciencia Animal, Universidad de Lleida, Av. Rovira Roure 191, 25198 Lleida, Spain; lauramartiarellano95@gmail.com; 2 Departamento de Producción Animal y Ciencia de los Alimentos, Facultad de Veterinaria, Instituto Agroalimentario de Aragón, Universidad de Zaragoza, 50013 Zaragoza, Spain; malatorr@unizar.es

**Keywords:** feed intake, pre-weaning survival, swine

## Abstract

**Simple Summary:**

A strong belief characterizes the choice of the feeding curve used during the transition sow period from gestation to lactation. Stepped feeding schedules are normally applied in this period, but ad libitum fed sows may have better post-partum performance. An ad libitum feeding strategy of sows from day −5 to +5 of lactation evidenced greater voluntary feed intake than the stepped schedule from two days before to five days post-farrowing. However, ad libitum feeding during the peri-partum did not show any clear advantage on the piglet survival at farrowing or post-partum reproductive recovery of sows.

**Abstract:**

This study hypothesized that the ad libitum feeding of sows during the peri-partum may increase the neonatal survival of the piglets and the post-partum recovery of the sow. The aim of the study was to evaluate the effects of two peri-partum feeding strategies (ad libitum and stepped from day 110 of gestation to day 5 post-partum) on the feed intake, the reproductive performance of sows, as well as the survival of their piglets. A total of 90 Landrace x Large-White dams sired by Duroc were used. The sows were randomly assigned to feeding treatments by balancing body condition and parity between groups. The feed intake of the sows in the peri-partum was lower in the stepped than in the ad libitum strategy. The increase of the feeding level in the ad libitum sows was not counterbalanced by higher number of weaned piglets or shorter weaned to oestrus interval.

## 1. Introduction

The peri-partum feeding strategy may affect subsequent piglet survival and sow performance, but there is no general agreement for the best practice. A mild transition from gestation to lactating metabolism is essential to attain a good productive performance during the sow post-partum [1]. Colostrum production increases during the last week of gestation [2], and subsequent milk yield depends on genetics potential but also on feed intake [3].

Stepped feeding strategies before and after farrowing are common in Europe to reduce the post-partum hypogalactia syndrome [4], and because high feeding levels during late gestation have been previously described as risk factors for peri-partum hypophagia [5]. However, restricted feeding during the peri-partum may also reduce the overall feed intake during the lactaction period [6] and may increase the weaning to oestrus interval [7]. In this trade-off, a negative energy balance during the last days of pregnancy may improve the milk yield during the first week of lactation [8]. The aim of this study was to evaluate the effects of ad libitum feeding during the peri-partum on sow feed intake and reproductive performance.

## 2. Materials and Methods

This experiment was conducted in a commercial farm in North-Eastern Spain (Alcarrás, Lleida, Spain), between January and February 2019. A total of 90 crossbred TN70 sows (Topigs Norsvin, Madrid, Spain), from two consecutive weekly batches, were randomly divided in two groups; ad libitum or stepped feeding in two meals (7:00 and 17:00 h) from day 110 of gestation to day 5 of lactation (n = 45 per batch and dietary treatment). The body condition score (BCS) of sows was assessed on a 1 to 5 scale picture [9] soon after the sows’ arrival at the farrowing room to balance the thin sows (BCS = 2) between treatments (32.6% vs. 20.5% of thin sows in ad libitum and stepped, respectively, *p* > 0.05). In addition, parity number was taken into account when assigning the treatments (4.4 ± 0.18 vs. 4.2 ± 0.17 in ad libitum and stepped, respectively, least square mean ± standard error; *p* > 0.05). The TN70 hybrid sows is a prolific female line with good weaning abilities and with an outstanding contribution to finishing performance [10]. The sows had been sired by Duroc boars (DanBred, Copenhagen, Denmark) to improve meat quality of the progeny. The welfare of sows was in accordance with the European Community standards (Council Directive 2008/120/EC). 

The scheduled stepped feeding curve supplied 3 kg of feed/day for the first 30 days of gestation, 2.5 kg/day from day 31 to 90 of gestation, and 3 kg/day from day 91 of gestation up to the pre-farrowing day. With the aim of avoiding feed wastage, the days before farrowing, the feed supply was gradually reduced to 1 kg/day (or lower, depending on sow appetite). After farrowing, the feed supply was increased by 1 kg/day (if feed was totally consumed in previous meal) up to a threshold of 10 kg/day, depending on the individual feed intake pattern of each sow. In contrast, ad libitum fed sows received an additional amount of 0.7 kg per meal (up to 1.4 kg/day higher when considering the two meals) from day 110 of gestation to day 5 of lactation compared to the sows fed the stepped schedule. The sows received a gestation feed from insemination to moving to the farrowing room (day 110 of pregnancy). During the experiment, the sows consumed a commercial lactation feed until weaning (from day 110 of pregnancy to day 25 of lactation). The feed nutrient composition is shown in Table 1. The feed was supplied in mash form, avoiding excessive water supply in the feeder to avoid feed waste. After the 5th day post-partum, both groups received the same stepped feed supply until the end of lactation in three meals (7:00, 12:00, and 17:00 h). During the experiment, the farrowing room was kept at 18–25 °C.

The feed was delivered by individual feed dispensers (Dos7, Collinson & Co Ltd., Lancashire, UK) that were adjusted volumetrically in each meal by the same operator. Before starting the data collection, the weight of each volume mark was registered in order to calculate the actual feed density in each line feeder. The feed supply was controlled twice daily (7:00 and 17:00 h) from day 110 of gestation to day 5 of lactation, and three times daily (7:00, 12:00, and 17:00 h) at day 23 of lactation. 

On the expected pre-farrowing day, the sows (but not gilts) were administered a prostaglandin analogue to induce farrowing (D-Cloprostenol, Galapan©, Barcelona, Spain). The sows showing a slow or dystocic farrowing were manually assisted and/or were supplied oxytocin (Facilpart©, León, Spain). 

Rectal temperature of sows was measured between 7:00 and 8:00 h on the day before, during and after farrowing to assess hyperthermia (≥39.5 °C) during the peri-partum. The alive piglets were kept with their dams during at least 12 h to assure colostrum consumption. Thereafter, they were cross-fostered within each experimental group (target 12–13 piglets/litter). The litters received creep feeding from day 10 of age onwards.

After farrowing, the reproductive performance of sows was registered (between 7:00 and 8:00 h): Number of piglets born, stillborn piglets, and mummified piglets before cross-fostering. The number of weaned piglets and sow weaning to oestrus interval were also registered. Post-weaning oestrus of sows was detected visually twice a day (08:00 and 16:00 h) in response to back-pressure with the presence of a teaser boar.

The data from three ad libitum fed sows and one-stepped fed sow were removed from statistical analyses due to incidences in data collection or feeding device problems. Three balanced parity classes were established according to the herd age structure: 1st–2nd parity, n = 24 sows; 3rd–5th parity, n = 40 sows; and 6th–10th parity, n = 22 sows. Data was analysed with the JMP statistical software (14th version, SAS Institute, Cary, NC, USA). Before applying linear modelling, data were checked for normality and homoscedasticity. To understand the effect of peri-partum days, feeding strategy and parity on the sow feed intake and rectal temperature, we performed a mixed model, considering pre- and post-partum days (−5, −4, −3, −2, −1, 0, 1, 2, 3, 4, 5, and 23, as an ordinal variable), feeding strategy (ad libitum, and stepped), parity (1st–2nd parity, 3rd–5th parity, and 6th–10th parity) and their single interactions as fixed effects, and the sow as a random effect, since repeated measures were collected on the same animal. To account for temporal autocorrelation of observations, calculations of the standard error (SE) and degrees of freedom were based on the Kenward–Roger method.

The body condition score of sows was classed as thin (BCS = 2, n = 23) or good (BCS of 3 or 4, n = 63), as no extreme BCS (1 or 5 score) were measured. The BCS of 3 and 4 was considered in a single group because only 14 out of the 86 sows (16.2%) were graded with a BCS of 4 (and all of them were ≥5th parity sows), as the industry feeding plan during gestation guaranteed a rather steady body condition across the sows. Hence, this grouping only aimed at identifying slightly emaciated sows (BCS = 2) from the rest. To ascertain the role of the feeding strategy, the parity, and the BCS group on the sow productive performance, we performed a standard least square model that considered the feeding strategy, the parity, and the BCS group as fixed effects, as well as their single interactions. The difference between means was evaluated with a Tukey test. The level of significance was set at 0.05. The results are expressed as least square means and their associated SE. The association between the feeding strategy and the proportion of sows showing hyperthermia (≥39.5 °C) and the proportion of sows requiring farrowing assistance were evaluated with contingency tables and Pearson test.

## 3. Results

There was no interaction between feeding strategy and parity (*p* > 0.05), nor between feeding strategy and BCS group (*p* > 0.05). Therefore, the effect of feeding strategy on the response variables is described separately.

**Figure 1:**
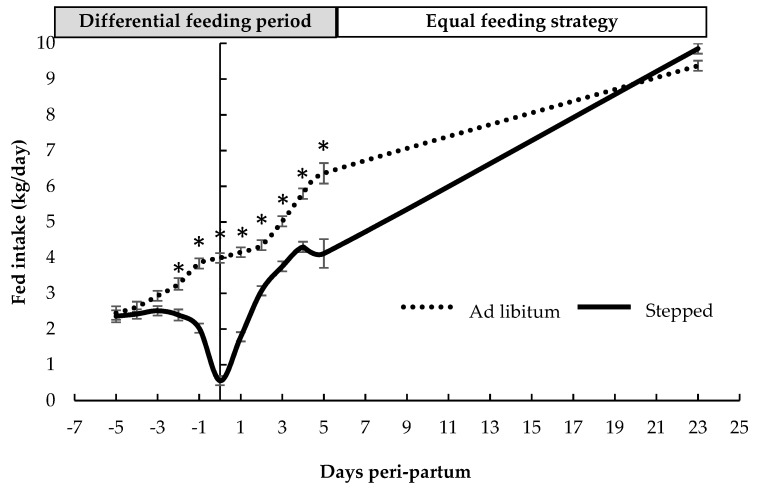
Sow feed intake during the peri-partum period according to ad libitum and stepped (pre-established) feeding strategy irrespective of parity number and body condition score (least square mean ± standard error). The time interval with asterisks show significant differences between treatments (intercept for the whole peri-partum = 3.9 ± 0.07 kg/day, slope for ad libitum treatment = +0.6 ± 0.07 kg/day; *p* < 0.05).

The sow feed intake in both stepped and ad libitum feeding strategies during the peri-partum is shown in Figure 1. There were no differences between feeding strategies from day −5 to day −3 of lactation (*p* > 0.05) but the sow feed intake from day −2 to day +5 of lactation was nearly half on average in the stepped than in the ad libitum feeding strategy (2.8 ± 0.18 vs. 4.6 ± 0.16 kg/day, respectively, *p* < 0.05). From day 6 post-partum, feed consumption was similar in both groups and the lack of differences was maintained until the end of lactation (day 23) (9.9 ± 0.14 vs. 9.4 ± 0.14 kg/day, in stepped and ad libitum, respectively, *p* > 0.05).

Sow rectal temperature increased gradually from the previous day of farrowing (37.7 ± 0.06, 38.4 ± 0.06, and 38.9 ± 0.06 °C in the pre-, during and post-farrowing day, respectively, *p* < 0.05). However, this variable was not different between feeding strategies in any time point (average of the three time points was 38.3 ± 0.05 °C in stepped and 38.3 ± 0.05 °C in ad libitum, respectively; *p* > 0.05). Hyperthermia was not detected in any sow the day before farrowing, but it was indeed measured in 7 out of 86 sows (8.1%) on the farrowing day and 15 out of 86 sows (17.4%) on the day after farrowing, without differences across feeding strategies (*p* > 0.05). 

The reproductive performance of the sows did not differ between feeding strategies (Table 2, *p* > 0.05). The BCS group of sows did not affect the feed intake, hyperthermia, or farrowing assistance (*p* > 0.05). However, thin sows resumed oestrus later than sows in good body condition (5.2 ± 0.30 vs. 4.3 ± 0.19 days post-weaning, respectively; *p* < 0.05).

## 4. Discussion

This study was designed to evaluate the effects of ad libitum feeding during the peri-partum on sow feed intake and reproductive performance. The main finding was that actual sow feed intake during the days around farrowing doubled the scheduled feeding curve supplied, although no differences were observed in the litter survival or the subsequent sow reproductive performance.

A strong belief rather than scientific evidence characterizes the choice of the feeding curve used during the transition sow period from gestation to lactation [11]. However, these authors appointed that the appetite of sows seemed to be a limiting factor for nutrient intake when the feed supply is increased in early lactation and therefore it is common to feed sows below their energy requirement. Restricted feeding in peri-partum sows may be of interest because this leads to a reduction in faecal output that decrease the cleaning needs behind the farrowing crates, although then the sows can suffer a greater risk for constipation [12]. The estimated energy requirements of transition multiparous sows from day 105 to 115 of gestation is approximately 39 MJ/day of metabolizable energy (ME) [13]. In this case, they should have consumed 3 kg/day of the current feed (13 MJ ME/kg) to meet their energy requirements, which was not attained in the stepped feeding strategy. Thereafter, the energy requirements increase substantially from day 2 of lactation onwards, due to a greater milk production [14], up to a range between 75 and 84 MJ ME/day at the end of first week of lactation, depending on parity, litter size, and gain [15,16]. This would mean that early lactating sows should consume between 5.8 and 6.5 kg/day of the current feed to meet their energy requirements at the end of the first week post-partum. Indeed, this was only attained in the ad libitum feeding strategy, although the sows fed the stepped strategy reached similar feed intake to their counterparts at the end of lactation. 

Increasing moderately the feeding level at the end of gestation, i.e., 1.8 vs. 2.2 kg/day, has been negatively correlated with the post-partum appetite [17], but this lactating hypophagy was not observed in other high feeding level conditions at the end of gestation, i.e., 2.6 vs. 3.3 kg/day [18], 1.5 vs. 4.0 kg/day [19], 3.0 vs. 7.0 kg/day [20]. The previous differences among studies may be due to different feed energy content, which is normally greater in American rationing than in European standards. In this study, the ad libitum fed sows showed greater appetite, at least, the week before and after farrowing, whereas this group was balanced with step-fed sows at the end of lactation. 

In loosed-housed conditions, a step-up strategy with gradual increase of feed supply was also recommended over ad libitum feeding, that always involved small amount of left over feed in the trough after each feeding [21], but in such situations the feeding curves must be frequently revisited to take into account the different environmental variation factors. According to Solà-Oriol and Gasa [22], the optimal feed intake during lactation may be a question of compromise between increasing feed on offer and avoiding marked feed drops. 

The ad libitum feeding strategy during the peri-partum had no detrimental effects on sow rectal temperature or farrowing assistance requirements. This is not in accordance with Neil et al. [23], who observed that ad libitum fed sows had greater rectal temperature and post-partum agalactia than step-wise fed sows until three days post-partum, while Neil [24] declared no advantage in delaying the introduction of ad libitum feeding until three days after farrowing because it did not decrease piglet mortality or post-partum agalactia.

In this study, the weaning to oestrus interval of sows did not differ between stepped and ad libitum feeding strategies. This similar reproductive response across feeding strategies may be associated with a counterbalanced appetite pattern in the step-fed sows, that increased their feed intake from day 5 to 23 of lactation to attain similar outcome to the ad libitum fed sows. This adaptive response was also observed by Decalawe et al. [19]. In agreement, stair-stepped compared to ad libitum feeding pattern during the first week of lactation (3.3 vs. 5.0 kg/day) did not increase the weaning to oestrus interval either [25].

In conclusion, the ad libitum feeding strategy of sows in the farrowing room evidenced greater voluntary feed intake than the stepped schedule from two days before to five days post-farrowing. However, ad libitum feeding during the peri-partum did not show any clear advantage on piglet survival at farrowing or post-partum reproductive recovery of sows.

## Figures and Tables

**Table 1:** Calculated nutrient composition of feed used during the peri-partum and lactation.

Nutrient	
Net energy (MJ/kg)	10
Ether extract (%)	5.5
Starch (%)	34.0
Crude Fibre (%)	5.0
Neutral-detergent Fibre (%)	18.9
Ash (%)	6.4
Crude Protein (%)	17.5
Standardized Ileal Digestible (SID) Lysine (%)	0.81
SID Methionine (%)	0.24
SID Methionine+Cysteine (%)	0.48
SID Threonine (%)	0.57
SID Tryptophan (%)	0.17
SID Valine (%)	0.67
SID Isoleucine (%)	0.57
Calcium (%)	1.0
Phosphorus (%)	0.6
Sodium (%)	0.2
Electrolyte balance (Na+K-Cl) (mEq/kg)	242.2
Vitamin A (IU/kg)	9500
Vitamin D (IU/kg)	1500
Vitamin E (IU/kg)	60

**Table 2:** Sow performances according to the peri-partum feeding strategy irrespective of parity number and body condition score (least square mean ± standard error).

	Stepped	Ad Libitum	*p*-Value ^1^
n	44	42	-
Total born piglets (number)	13.5 ± 0.44	13.6 ± 0.47	NS
Stillborn piglets (%)	4.2 ± 1.34	3.3 ± 1.44	NS
Mummified piglets (%)	1.8 ± 1.09	2.1 ± 1.17	NS
Farrowing-assisted sows (%)	65.9	52.4	NS
Piglets after fostering (number)	12.6 ± 0.13	12.8 ± 0.14	NS
Weaned piglets (number)	11.5 ± 0.09	11.4 ± 0.10	NS
Sow weaning to oestrus interval (days)	4.9 ± 0.24	4.6 ± 0.26	NS

^1^ NS = non-significant (*p* > 0.05).
